# Estado de salud y barreras en la atención de niños con defectos congénitos nacidos entre el 2011 y el 2017 en dos instituciones de salud de Cali

**DOI:** 10.7705/biomedica.4906

**Published:** 2020-03-30

**Authors:** Luisa F. Imbachí, Lina M. Ibáñez, Paula Hurtado-Villa

**Affiliations:** 1 Grupo de Investigación en Ciencias Básicas y Clínicas de la Salud, Departamento de Ciencias Básicas de la Salud, Facultad de Ciencias de la Salud, Pontificia Universidad Javeriana, Cali, Colombia Pontificia Universidad Javeriana Departamento de Ciencias Básicas de la Salud Facultad de Ciencias de la Salud Pontificia Universidad Javeriana Cali Colombia; 2 Centro Médico Imbanaco, Cali, Colombia Centro Médico Imbanaco Cali Colombia

**Keywords:** anomalías congénitas, vigilancia epidemiológica, pediatría, mortalidad infantil, estudios de seguimiento, Congenital abnormalities, epidemiological monitoring, pediatrics, infant mortality, follow up studies

## Abstract

**Introducción.:**

Los defectos congénitos afectan entre el 2 y el 3 % de los recién nacidos, y son una carga importante entre las causas de morbilidad y mortalidad infantil en los primeros cinco años de vida. En Colombia, fueron la segunda causa de mortalidad infantil según los reportes del Departamento Administrativo Nacional de Estadística (DANE) para el 2011.

**Objetivo.:**

Describir el estado de salud y las barreras en la atención de niños con defectos congénitos nacidos entre el 2011 y el 2017 en dos instituciones de salud de Cali.

**Materiales y métodos.:**

Se hizo un estudio observacional descriptivo de corte transversal. Se incluyeron los nacidos entre enero de 2011 y diciembre de 2017 con, al menos, un defecto congénito, a quienes se les hizo seguimiento telefónico.

**Resultados.:**

De 54.193 nacidos en el período analizado, 1.389 (2,56 %) tenían, por lo menos, un defecto congénito. Todos los casos se clasificaron según la escala de pronóstico y se incluyeron 881 en el seguimiento. El defecto congénito más frecuente fue la malformación congénita cardíaca, con 88 (9,99 %) casos; en segundo lugar, las malformaciones o defectos del riñón, con 73 (8,29 %) casos; en tercer lugar, el síndrome de Down, con 72 (8,17 %) casos, y en cuarto lugar, las anormalidades testiculares, con 56 (6,36 %). Ciento sesenta y uno (35,46 %) de los cuidadores de los 454 casos con seguimiento efectivo, manifestaron haber encontrado, por lo menos, un tipo de barrera en la atención.

**Conclusión.:**

Se deben implementar programas de seguimiento de los pacientes con defectos congénitos, que contribuyan a disminuir la morbimortalidad.

## Abstract (en)

**Introduction:**

Birth defects affect 2-3% of births contributing an important load to the causes of infant morbidity and mortality during the first five years of life. In Colombia, they are the second cause of infant mortality according to reports from the *Departamento Administrativo Nacional de Estadística* (DANE) 2011.

**Objective:**

To describe the state of health and barriers in the health care of children with congenital defects born between 2011 and 2017 in two institutions of Cali.

**Materials and methods:**

We conducted a descriptive cross-sectional observational study. We included babies born between January, 2011, and December, 2017, with at least one congenital defect who were followed up by telephone.

**Results:**

Out of 54,193 births during the period, 1,389 (2.56%) newborns had at least one congenital defect. All cases were classified according to the prognostic scale and 881 were included in the follow-up. The most frequent congenital defect was congenital cardiac malformation with 88 cases (9.99%), followed by malformation/defect of the kidney with 73 cases (8.29%), Down syndrome with 72 cases (8.17%), and testicular abnormalities with 56 cases (6.36%). Out of the 454 cases with effective follow-up, 161 (35.46%) of the caregivers stated that they had experienced at least one type of barrier during health care.

**Conclusion:**

Follow-up programs should be implemented for patients with birth defects to contribute to reducing morbidity and mortality.

Los defectos congénitos, trastornos o malformaciones estructurales o funcionales, afectan a entre 2 y 3 % de los recién nacidos ^1^^,^^2^, lo que representa una carga importante entre las causas de morbilidad y mortalidad infantil en los primeros años de vida. En Latinoamérica ^3^ y en Colombia, son la segunda causa de mortalidad ^4^.

El impacto sobre la morbilidad se relaciona con la cronicidad, la ausencia de tratamiento efectivo, las comorbilidades congénitas asociadas o adquiridas y la discapacidad, cuya atención y pronóstico exigen equipos multidisciplinarios ^5^. En términos de años de vida ajustados por discapacidad (AVAD), los defectos congénitos representan entre 25,3 y 38,8 millones de AVAD y ocupan la posición 17 entre las causas de carga de enfermedad en todo el mundo. Dicha situación sería evitable en 57 % (12 millones) de los casos, si las facilidades quirúrgicas de los países de medianos y bajos ingresos fueran las mismas de aquellos desarrollados ^6^. A nivel nacional, los datos sobre la morbilidad por esta causa son escasos.

En Colombia, el Programa de Vigilancia de Malformaciones Congénitas de Bogotá registra información y hace seguimiento de los pacientes desde el 2001 ^7^ con base en la metodología del Estudio Colaborativo Latinoamericano de Malformaciones Congénitas (ECLAMC) ^8^, programa que se viene implementando en Cali desde octubre de 2010. A partir de los registros poblacionales de defectos congénitos, se ha optimizado la información sobre su prevalencia ^5^ y, con el seguimiento, se busca mejorar el pronóstico de la discapacidad asociada mediante la verificación del estado de salud de los pacientes, así como de su relación con los servicios de salud, para favorecer así el desarrollo futuro de procesos que tengan un impacto positivo en la salud de la población ^9^, incluida la detección de pacientes con necesidades especiales, con el fin de garantizar para ellos y su familia la remisión apropiada a los servicios requeridos ^10^; finalmente, esto tendrá un efecto positivo en la mortalidad y la morbilidad infantil relacionadas ^11^.

La Sociedad Italiana de Enfermedades Genéticas Pediátricas y Discapacidad propone intervenciones integrales, de carácter personalizado, incluidos los aspectos globales, los derechos y necesidades de salud, y los aspectos clínicos con intervenciones sociales, de rehabilitación y formativas para el paciente y su familia ^12^. En el país, la Constitución Política define la vida, la integridad física, la salud y la seguridad social, como derechos fundamentales de los niños para permitir el desarrollo de sus capacidades ^13^. En este contexto, en el estudio se propuso describir el estado de salud y las barreras en la atención de niños con defectos congénitos, nacidos entre el 2011 y el 2017 en dos instituciones de salud de Cali.

## Materiales y métodos

### Tipo de estudio

Se hizo un estudio observacional descriptivo y de corte transversal.

### Área de estudio

Cali es la capital del departamento del Valle del Cauca (Colombia) y es la tercera ciudad más poblada del país, con 2,4 millones de habitantes. La vigilancia de defectos congénitos se lleva a cabo en dos instituciones hospitalarias de tercer nivel de atención de la ciudad.

### Población de estudio

Se incluyeron los casos del Programa de Vigilancia y Seguimiento de Defectos Congénitos de la Pontificia Universidad Javeriana de Cali, captados durante el período de enero de 2011 a diciembre de 2017, lapso en el que se hizo la vigilancia de 54.193 nacimientos en dos clínicas privadas de Cali y se detectaron 1.389 recién nacidos con un defecto congénito, por lo menos.

Todos los casos se clasificaron según la escala de pronóstico propuesta por Fernández, *etal.*^14^, y se incluyeron 881 en el análisis.

### Recolección de la información

La información se tomó de la base de datos del programa. Se llamó a los cuidadores de los pacientes seleccionados para el análisis entre marzo de 2014 y marzo de 2018. Las llamadas se hicieron durante varios días y a diferentes horas para poder completar el cuestionario e incrementar las tasas de respuesta. La frecuencia de las llamadas se ajustó según cada caso y de acuerdo con los planes de seguimiento de pacientes por tipo de defecto congénito y sus consecuencias, propuesto en el Programa de Seguimiento de Niños con Anomalías Congénitas de Bogotá ^7^.

Durante la llamada, se diligenciaba un cuestionario sobre la situación de salud del niño, información que se registró en un archivo de Excel. El estado de los pacientes se definió como la relación entre sus problemas de salud y su contexto social y familiar en una fecha determinada, es decir, una descripción del paciente en relación con estos aspectos.

Las variables incluidas fueron las siguientes: grupo de malformaciones congénitas; resultado de las llamadas clasificadas como efectivas (cuando se lograba comunicación con alguno de los cuidadores del niño y se obtenía información del estado y la atención en salud del niño e información general de la madre y del niño), y no efectivas (aquellas en las que no se lograba establecer una comunicación con la madre o cuidador del niño o niña); clasificación del estado de salud del niño en las siguientes categorías: estable con la malformación (en el momento del seguimiento no hay riesgo para el niño), mejoría total (cuando se observa una mejoría espontánea al tratarse de defectos menores o por intervención quirúrgica), sin la malformación (casos en los que el cuidador niega algún tipo de malformación con base en la información del personal de salud que ha estado en contacto con el niño), corrección quirúrgica (casos en que se ha practicado algún procedimiento quirúrgico para la mejoría o estabilización del niño), aparición de complicaciones o efectos secundarios (casos en que ha habido hospitalización o se han presentado otros problemas de salud), muerte (casos en que en, el momento del seguimiento, el niño ha fallecido a causa de la malformación o por efectos secundarios) y barreras en la atención de salud.

### Análisis de datos

Se hizo un análisis descriptivo de las variables y se resumieron en tablas de frecuencias. Toda la información se registró en Excel y se analizó en el programa estadístico Stata 14,0™ (Stata Corp, 2014, College Station, TX, USA).

### Consideraciones éticas

Las madres de los recién nacidos con defectos congénitos registrados en el programa de vigilancia firmaron un consentimiento informado de participación en el programa después de que se les explicaron los objetivos de la vigilancia, así como para recibir llamadas telefónicas posteriores de seguimiento.

Según la Resolución N° 008430 del 4 de octubre de 1993 emitida por el Ministerio de Salud de la República de Colombia, que establece las normas científicas, técnicas y administrativas para la investigación en salud, este estudio contribuye al conocimiento de los vínculos entre las causas de la enfermedad, la práctica médica y la estructura social, y en él prevalece el criterio del respeto a la dignidad y la protección de los derechos y el bienestar del ser humano y se protege la privacidad del individuo sujeto de investigación.

Se trata de una investigación sin riesgo por emplear técnicas y métodos de investigación documentales retrospectivos y porque no se realizó ninguna intervención ni modificación intencionada de las variables biológicas, fisiológicas, sicológicas o sociales de los individuos participantes. Entre dichas técnicas, se cuentan la revisión de historias clínicas, las entrevistas, los cuestionarios y otras que no implican el manejo de aspectos sensibles de la conducta.

## Resultados

En el periodo de estudio, de los 1.389 recién nacidos con al menos un defecto congénito, se incluyeron en el análisis 881 casos después de clasificarlos utilizando la escala de pronóstico. La mayoría de los casos (560; 63,56 %) se clasificó como Ilb, es decir, malformaciones congénitas con riesgo de mortalidad o grave discapacidad (II), en las que la intervención en salud mejora al paciente hasta la normalidad o este empeora gravemente (b).

De los 881 recién nacidos seleccionados, 541 (61,41 %) eran de sexo masculino y 690 (78,32 %) procedían de Cali; el porcentaje restante se distribuía en municipios de los departamentos del Valle del Cauca y del Cauca, cercanos a esta ciudad.

En el cuadro 1 se presentan los defectos congénitos más frecuentes por subgrupos. En primer lugar, se encuentra la malformación congénita cardíaca con 88 (9,99 %) casos; en segundo lugar, la malformación o defecto del riñón con 73 (8,29 %); en tercer lugar, el síndrome de Down con 72 (8,17 %); en cuarto lugar, las anormalidades testiculares con 56 (6,36 %) y, en quinto lugar, las hipospadias con 51 (5,79 %). Todas las malformaciones con una frecuencia menor de 1,00 % se resumieron en una sola categoría (otras).

**Cuadro 1:** Clasificación según tipo de malformación

Subgrupos	n	%
Malformación congénita cardiaca	88	9,99
Malformación o defecto del riñón	73	8,29
Sfndrome de Down	72	8,17
Anormalidades testiculares	56	6,36
Hipospadias	51	5,79
Talipes	47	5,33
Defecto obstructivo vésico-uretero-renal	39	4,43
Labio leporino con paladar hendido o sin él	28	3,18
Anormalidad de la cadera	23	2,61
Malformación o defecto de maxilares	23	2,61
Anormalidades escrotales	19	2,16
Paladar hendido	19	2,16
Malformación congénita inespecffica de la cara o el cuello	18	2,04
Microtia	16	1,82
Malformaciones congénitas múltiples	14	1,59
Microcefalia	14	1,59
Atresia o estenosis esofágica	12	1,36
Genitales externos ambiguos o ausentes	12	1,36
Polidactilias	12	1,36
Anormalidad por reducción de miembros	11	1,25
Deformidad o malformación de la oreja	11	1,25
Hidrocefalia	11	1,25
Malformación o deformidad de la cabeza	11	1,25
Cerebro	9	1,02
Defecto o malformación de la pared abdominal	9	1,02
Defecto o malformación de otros órganos abdominales	9	1,02
Hemangioma	9	1,02
Otras	165	18,73
Total	881	100,00

Con respecto al seguimiento, no se pudieron contactar 427 (48,47 %) cuidadores de los pacientes debido a: celulares apagados (125: 14,19 %), porque no contestaron (94: 10,67 %), estaban fuera de servicio (93: 10,56 %), número equivocado (47: 5,33 %), contestaban pero no se encontraba el cuidador (33; 3,75 %), sin número de contacto (22: 2,50 %) o número mal registrado (13: 1,48 %). El número promedio de marcaciones fue de 5,12, con un mínimo de 0 y un máximo de 16 marcaciones.

Se realizaron 454 (51,53 %) seguimientos efectivos (cuadro 2). El número promedio de marcaciones fue de 2,15, con un mínimo de 1 y un máximo de 12 marcaciones, y la duración promedio de cada llamada fue de 20 minutos.

**Cuadro 2:** Resultado del seguimiento según la clasificación del número de contacto

		Seguimiento efectivo			
Clasificación del número de contacto	Total no efectivo n (%)	Fallecidos n (%)	Vivos n (%)	Total efectivo n (%)	Total n (%)
Celular	258 (29,28)	32 (3,63)	263 (29,85)	294 (33,37)	553 (62,77)
Fijo	122 (13,85)	11 (1,25)	111 (12,60)	122 (13,85)	244 (27,70)
Mixto	12 (1,36)	4 (0,45)	33 (3,75)	37 (4,20)	49 (5,56)
Número mal registrado	13 (1,48)	0 (0,00)	0 (0,00)	0 (0,00)	13 (1,48)
Sin número de contacto	22 (2,50)	0 (0,00)	0 (0,00)	0 (0,00)	22 (2,50)
Total	427 (48,47)	47 (10,35)	407 (89,65)	454 (51,53)	881 (100,00)

En cuanto al régimen de afiliación a la seguridad social, 345 (75,99 %) de los casos con seguimiento efectivo pertenecían al régimen contributivo. La clasificación del estado de salud de los niños se describe en el cuadro 3: 193 (42,51 %) continuaban presentando la malformación, pero estaban estables, es decir, en el momento del seguimiento no había riesgo para el niño.

**Cuadro 3:** Clasificación del estado de salud de los niños con seguimiento efectivo

Clasificación del estado de salud	n	%
Estable con la malformación	193	42,51
Mejorfa total	61	13,44
Sin la malformación	54	11,89
Muerto	47	10,35
Corrección quirúrgica	34	7,49
Aparición de complicaciones o efectos secundarios	25	5,51
Estable con la malformación; corrección quirúrgica	21	4,63
Corrección quirúrgica; aparición de complicaciones o efectos secundarios	13	2,86
Estable con la malformación; aparición de complicaciones o efectos secundarios	3	0,66
Sin información	2	0,44
Estable con la malformación; corrección quirúrgica; aparición de complicaciones o efectos secundarios	1	0,22
Total	454	100,00

En el momento del seguimiento, en 47 (10,35 %) de los 454 casos con seguimiento efectivo, el paciente había fallecido (cuadro 2) debido a la condición misma o a complicaciones propias del defecto congénito u otras que no habían sido claras según lo referido por los cuidadores.

De los 454 casos con seguimiento efectivo, en 161 (35,46 %), los cuidadores manifestaron haber encontrado, por lo menos, un tipo de barrera durante el proceso de atención en salud del niño. En el cuadro 4 se describen las barreras de atención agrupadas por año y según la información brindada por los cuidadores. Las barreras más frecuentes tuvieron que ver con las autorizaciones y la oportunidad de las citas médicas. Por último, los años en que los cuidadores reportaron más barreras fueron el 2015 y el 2017, con 32 (19,88 %) y 33 (20,50 %), respectivamente.

**Cuadro 4:** Barreras de atención en la salud reportadas por los cuidadores desde 2011 hasta 2017

Barrera en la atención	2011 n (%)	2012 n (%)	2013 n (%)	2014 n (%)	2015 n (%)	2016 n (%)	2017 n (%)	Total n (%)
Demora en agendar citas	0 (0,00)	7 (4,35)	5 (3,11)	9 (5,59)	3 (1,86)	2 (1,24)	2 (1,24)	28 (17,39)
Dificultad para acceder a citas	3 (1,86)	2 (1,24)	2 (1,24)	4 (2,48)	8 (4,97)	3 (1,86)	2 (1,24)	24 (14,91)
Dificultades administrativas	0 (0,00)	0 (0,00)	2 (1,24)	3 (1,86)	2 (1,24)	1 (0,62)	7 (4,35)	15 (9,32)
Dificultades con las autorizaciones	3 (1,86)	6 (3,73)	7 (4,35)	6 (3,73)	4 (2,48)	11 (6,83)	10 (6,21)	47 (29,19)
Dificultades de afiliación	0 (0,00)	0 (0,00)	0 (0,00)	3 (1,86)	6 (3,73)	2 (1,24)	4 (2,48)	15 (9,32)
Mala atención	0 (0,00)	0 (0,00)	0 (0,00)	3 (1,86)	4 (2,48)	6 (3,73)	4 (2,48)	17 (10,56)
Mala información	0 (0,00)	1 (0,62)	0 (0,00)	0 (0,00)	4 (2,48)	0 (0,00)	3 (1,86)	8 (4,97)
Problemas con medicamentos	0 (0,00)	0 (0,00)	0 (0,00)	0 (0,00)	0 (0,00)	3 (1,86)	1 (0,62)	4 (2,48)
Problemas de desplazamiento	0 (0,00)	0 (0,00)	0 (0,00)	0 (0,00)	1 (0,62)	2 (1,24)	0 (0,00)	3 (1,86)
Total	6 (3,73)	16 (9,94)	16 (9,94)	28 (17,39)	32 (19,88)	30 (18,63)	33 (20,50)	161 (100,00)

## Discusión

La morbilidad relacionada con los defectos congénitos se establece por el pronóstico de discapacidad y por el efecto modificador que pueden tener sobre ellos las intervenciones en salud. La mayoría de los pacientes con defectos congénitos se clasificó en la categoría II en la escala de pronóstico descrita por Fernández, *et al.*^14^, siendo los subgrupos más frecuentes los de malformaciones congénitas cardíacas, seguido por las malformaciones o defectos del riñón, el síndrome de Down y las anormalidades testiculares. Esto, en general, es similar a lo reportado por otros estudios en nuestro país ^7^^,^^14^^-^^16^, aunque el orden de las posiciones cambia un poco. Los defectos congénitos son una causa importante de mortalidad y morbilidad infantil cada vez más evidente en Colombia gracias al control de las enfermedades infecciosas y al mejoramiento de las condiciones de higiene como parte del proceso de transición epidemiológica. Según datos de la Organización Mundial de la Salud (OMS), las anomalías congénitas causan 3,2 millones de discapacidades al año ^17^, lo que resulta de interés en salud pública.

En un porcentaje importante de los casos, no se logró establecer una comunicación efectiva (48,47 %) con los cuidadores de los niños debido, en parte, al registro incompleto o inadecuado de los datos en el formato del programa de vigilancia o al tiempo transcurrido desde la captación del caso hasta el contacto telefónico. De ahí la importancia del registro preciso de la información en los programas de vigilancia. Es así como el contacto efectivo se logró en 51,42 % de los casos, cifra importante para esta primera aproximación, lo que puede mejorar en el futuro, y similar a la efectividad del seguimiento telefónico reportado en el programa de vigilancia de Bogotá ^7^.

En cuanto al estado de salud, en la mayoría de los casos de pacientes con defecto congénito, estos se encontraban estables por tratarse de una condición crónica. Los casos de mortalidad se relacionaron directamente con el defecto congénito o con complicaciones asociadas.

Por otro lado, más de un tercio de los cuidadores de los pacientes con los que se logró establecer una comunicación efectiva manifestó haber encontrado algún tipo de barrera en la atención en salud (35,46 %), siendo las más frecuentes las relacionadas con dificultades para las autorizaciones, la demora en agendar citas y la dificultad para obtener una, lo que coincide con lo hallado por otros autores ^18^, aunque en ese otro estudio el conocimiento del sistema ocupó el primer lugar, el tiempo de espera, el segundo, la oferta de servicios, el tercer lugar y las barreras administrativas, el octavo lugar. El acceso a los servicios de salud es una preocupación mundial y, en Colombia, la legislación y la jurisprudencia han hecho más explícito el propósito de garantizar el acceso y han reconocido la salud como un derecho humano fundamental ^18^. A pesar de ello, algunos cuidadores manifestaron que tuvieron que interponer acciones de tutela para proteger sus derechos a la atención adecuada, situación que potencia el riesgo de complicaciones y el mal pronóstico de los defectos congénitos descritos.

El reporte de barreras por año muestra que, en general, este fue constante desde el 2014 hasta el 2017; la diferencia con los primeros años puede deberse principalmente a la captación de casos, ya que el programa estaba empezando.

En las llamadas efectivas, se detectó una carga emocional en el cuidador manifestada como ansiedad, sufrimiento, tristeza e impotencia relacionados con la situación de salud del niño, las dificultades económicas y de acceso a la atención que consideraban necesaria para el bienestar del niño, así como por las dudas relacionadas con los trámites administrativos para la autorización de los servicios de salud, lo cual puede ser un reflejo de la falta de acompañamiento integral a las familias y de orientación adecuada por parte de las empresas prestadoras de servicios de salud (EPS) desde el punto de vista administrativo. También, manifestaron su incomodidad e insatisfacción ante el hecho de que hubo personal relacionado con el cuidado de la salud de los niños que desconocía su condición de base, situación que, en ocasiones, se traducía en evasión en la atención o en una atención inadecuada.

En el análisis de Jaramillo-Mejía, *etal.* sobre los determinantes de la mortalidad infantil en Colombia ^19^, se documenta el papel del tipo de aseguramiento, la calidad de los servicios de salud, y su oportunidad y disponibilidad para la población infantil. En el estudio de Mackie, *et al.,* en Quebec, Canadá, se señala que la pérdida de pacientes pediátricos con cardiopatías congénitas en el seguimiento es un problema prevalente ^20^ y se plantea que la educación de los pacientes, los cuidadores y los proveedores de atención primaria puede contribuir a disminuir el problema.

En el contexto local, la información que se presenta permite una aproximación inicial y global al estado de salud actual de niños con defectos congénitos en Cali, a la oportunidad de la atención en los servicios de salud, a algunas limitaciones halladas en este proceso, y a las complicaciones y la mortalidad por esta causa, aunque reconociendo las limitaciones del estudio en cuanto no hubo una aproximación más objetiva a la situación de cada niño.

En Colombia, hay normas para la defensa de los derechos de los niños. El artículo 27 del Código de la Infancia y la Adolescencia (Ley 1098 de 2006) ^21^ define el derecho a la salud y establece que ninguna entidad prestadora del servicio de salud puede abstenerse de atender a un niño que requiera atención. En el documento se define la salud integral como "la prestación de todos los servicios, bienes y acciones, conducentes a la conservación o la recuperación de la salud de niños, niñas y adolescentes".

En dicho documento se definen los derechos de los niños con discapacidad y se específica que "los niños que presenten anomalías congénitas o algún tipo de discapacidad tendrán derecho a recibir atención, desde el diagnóstico, tratamiento especializado, rehabilitación y cuidados especiales en salud". Además, se debe proporcionar apoyo a las personas responsables de su cuidado ^22^.

En conclusión, el seguimiento de los niños con defectos congénitos ayuda a conocer su estado de salud, y aspectos determinantes como la oportunidad en la atención, las dificultades de las familias y la percepción de los cuidadores sobre dicha atención, entre otros, lo cual permitirá plantear estrategias que contribuyan a disminuir la carga de enfermedad en este grupo poblacional, en la población infantil en general y en las familias.

Con el estudio se espera llamar la atención, no solo de la comunidad científica, sino también del personal de salud y de la misma comunidad, para reconocer los defectos congénitos como causa de morbimortalidad infantil. El contar con programas de seguimiento para estos pacientes permite detectar las barreras en la atención en salud, así como las necesidades de los pacientes y sus familias, con el objetivo de abordarlas y proporcionar los servicios de salud necesarios para mejorar su pronóstico general, su calidad de vida y la de sus familias.
